# In Systemic Sclerosis, Anxiety and Depression Assessed by Hospital Anxiety Depression Scale Are Independently Associated with Disability and Psychological Factors

**DOI:** 10.1155/2013/507493

**Published:** 2013-07-29

**Authors:** Angela Del Rosso, Svetlana Mikhaylova, Marco Baccini, Ilaria Lupi, Marco Matucci Cerinic, Susanna Maddali Bongi

**Affiliations:** ^1^Department of Experimental and Clinical Medicine, Division of Rheumatology, Denothe Centre, Careggi Hospital (AOUC), University of Florence, Viale Pieraccini 18, 50139 Firenze, Italy; ^2^ASL 10 Physiotherapy Outpatient Clinic and School of Physiotherapy, University of Florence, Viale Pieraccini 18, 50139 Firenze, Italy

## Abstract

*Background*. Anxious and depressive symptoms are frequent in Systemic Sclerosis (SSc). Our objective is to assess their prevalence and association with district and global disability and psychological variables. *Methods*. 119 SSc patients were assessed by Hospital Anxiety Depression Scale (HADS). Clinical depression and anxiety were defined for HADS score cutoff ≥8. Patients were assessed for psychological symptoms (RSES, COPE-NIV), hand (HAMIS, CHFDS, fist closure, and hand opening) and face disability (MHISS, mouth opening), global disability, and fatigue (HAQ, FACIT). *Results*. Both depression and anxiety in SSc are 36%. Depressive patients with comorbid anxiety have higher HADS-D score than patients with depression only (*P* = 0.001). HADS-A and -D are positively correlated with global disability, hands and mouth disability, fatigue, self-esteem and avoidance coping strategy, and, only HADS-A, also with social support (*P* < 0.05). By multiple regression, HADS-D is independently associated with FACIT-F (*P* < 0.001), RSES (*P* < 0.001), and MHISS total score (*P* = 0.016), together explaining 50% of variance. HADS-A is independently associated with RSES (*P* = 0.006), COPE-NIV SA (*P* = 0.003), COPE-NIV SS (*P* = 0.008), FACIT-F (*P* = 0.022), and MHISS mouth opening (*P* = 0.029), explaining 41% of variance. *Conclusions*. In SSc depression and anxiety correlate to local and global disabilities and psychological characteristics. Depressive patients with comorbid anxiety have higher level of depressive symptoms.

## 1. Introduction

Systemic Sclerosis (SSc), characterized by skin, joint, and muscle modifications, leads to disability and impairs health related quality of life (QoL) [1].

As a consequence of chronicity, SSc patients report high level of pain, fatigue, and impaired physical functioning [2, 3]. SSc often disfigures the most visible and socially relevant parts of the body like the face and the hands. These changes may affect social relationships, cause functional problems, and alter patient's self-image, leading to body image dissatisfaction, thus reducing QoL [4, 5]. 

In addition to physical impairments and symptoms, SSc patients also report higher rates of anxiety and depression (ranging from 18% to 65%) than general population and other patients groups [6, 7]. 

In SSc, previous studies examining problems associated with depression and anxiety reported various different correlations. Depressive symptoms were related with reduction of working ability, social activity and capacity to respond to stressors, higher level of helplessness, pain, fatigue, social support, emotional focused coping, and fear of disease progression [8, 9]. Roca et al. reported depression as correlated to younger age, digital ulcers, and higher functional disability. Correlations between depression and aspects of personality, psychosocial adjustment to illness, and lack of social support [6] were also shown. In some studies, depressive symptoms were linked to SSc severity [10–12], pulmonary restrictive disease [13], fatigue [14], or other physical symptoms [10, 12]. Only one of them evaluated mood disorders according to disease subsets [15], not showing any difference. 

While anxiety has been largely studied in other chronic diseases and in rheumatoid arthritis (RA) [16, 17], only few studies addressed this problem in SSc patients [13, 15, 18–20], in which it was associated with education, joint deformity, disease activity, disease duration [21], and gender [19]. However, none of these studies evaluated the potential effects of self-esteem, mouth, and hand disability on anxiety and depression in SSc. 

Only few works evaluated mood disorders in SSc according to Hospital Anxiety and Depression Scale (HADS). Baubet et al. used HADS only as a screening tool for anxiety and depressive disorders in SSc patients then evaluated by Mini International Neuropsychiatric Interview [13]. Nguyen, assessing differences in HADS according to gender, did not evaluate correlation between HADS scores and other parameters [19]. 

The only study correlating HADS to psychological and clinimetric parameters by a multivariate regression analysis found fear of negative evaluation and overall interference of SSc on activities as the most important contributions to HADS-A and HADS-D, respectively, but not assessed disability [20]. 

Data from the literature consistently suggest that in the majority of subjects presenting both depressive and anxiety symptoms, defined as comorbid cases, anxiety disorders are primary to depression and that patients with anxiety are at higher risk to develop major depression [22]. Depressive patients with comorbid anxiety symptoms have a more difficult course, with a decreased or a delayed response to treatment [23]. Comorbidity of depression and anxiety, at the best of our knowledge, was never evaluated in SSc patients. 

Basing on all these data, a work aiming to systematically assess a large panel of clinimetric and psychometric tools and to individuate by multivariate regression analysis their contribution to HADS scores is still lacking. 

Thus, the objectives of the present study are to assess the prevalence of depressive and anxiety symptoms in SSc; to evaluate whether comorbid anxiety may lead to higher level of depression; to assess the potential differences between subsets; to evaluate the possible correlation of depression and anxiety with hands and face involvement and disability, global disability, fatigue, and psychological characteristics.

## 2. Patients and Methods

One hundred nineteen SSc patients, fulfilling ACR criteria [24] [14 men and 105 women; 74 with limited SSc (lSSc); and 45 with diffuse SSc (dSSc)) [25]; age: 59.46 ± 13.87 years; disease duration, calculated as the distance from the first symptom after Raynaud's Phenomenon: 10.74 ± 7.42 years], were consecutively enrolled from the Division of Rheumatology of the University of Florence. 

Patients signed a written informed consent; the procedures were conducted in accordance with the 1975/83 Helsinki Declaration, and the study was approved by local ethical committee. 

### 2.1. Psychological Assessment

*Hospital Anxiety and Depression Scale (HADS) *is a reliable and valid self-report questionnaire for identifying and quantifying psychological distress [26]. It is a self-rating scale consisting of 2 subscales: HADS-A (anxiety) and HADS-D (depression). HADS includes 7 questions for each dimension, whose scores range from 0 (no depression, no anxiety) to 21 (maximal depression or anxiety). 

HADS was also administered to 50 healthy controls, age and sex matched to SSc patients (4 men and 46 women, age 52.10 ± 9.0, not significantly different from SSc patients for male: female ratio and for age), enrolled by a general practitioner among the patients visiting his outpatients clinic, without rheumatic or chronic diseases. 

The evaluation was based on cutoff score ≥8 in HADS-A and HADS-D, as indicated in autoimmune diseases [26] and in general population [27].

*Rosenberg Self-Esteem Scale (RSES)* is used to assess global self-esteem [28].

*Coping Orientation to Problems Experienced-New Italian Version (COPE-NVI)* is utilized to assess coping, the use of skills and strategies adopted to face stressful and difficult events. COPE-NVI is a 60-item self-report questionnaire exploring 15 different types of coping, grouped in 5 large essentially independent dimensions: Social Support (SS), Avoidance Strategies (AS), Positive Attitude (PA), Problem Solving (PS), and Transcendent Orientation (TO) [29]. 

### 2.2. Assessment of Hand Disability and Mobility

*Hand Mobility in Scleroderma Test (HAMIS)* is a performance-based test, composed of 9 items, all related to movements that are part of daily occupations [30]. Validity of the Italian version of HAMIS in SSc patients was demonstrated [31]. 

*Cochin Hand Functional Disability Scale (CHFDS)* is a self-report questionnaire, measuring hand functional ability in performing 18 daily tasks of living, reliable and valid in patients with SSc. [32, 33]. *Fist Closure and Hand Opening*. Fist closure is valued as the distance between the fingertip of the third finger and tenar eminence when making a fist, and hand opening as the distance between the fingertip of the third finger, when extended, and the table. Both measures are reported in centimeters and as means of two consecutive measurements. 

### 2.3. Assessment of Face Disability and Mobility

The functional involvement of face and mouth is assessed by the Mouth Handicap in Systemic Sclerosis (MHISS) scale and mouth opening. 

*Mouth Handicap in Systemic Sclerosis Scale (MHISS) *assessing the handicap associated with mouth disability in SSc consists in 12 items (each scored 0–4, with a total score ranging from 0 to 48) divided into 3 subscales: subscale 1 examines handicap related to reduced mouth opening; subscale 2 assesses handicap related to sicca syndrome; subscale 3 examines aesthetic concerns [34, 35]. 

*Maximal Oral Aperture *is measured as the distance between the lower and upper lips vermilion line while the patient opens up mouth as widely as possible. The measure is reported in centimeters and as mean of two consecutive measurements.

### 2.4. Assessment of Global Disability and Fatigue

*Health Assessment Questionnaire (HAQ) *[36] is a self-report questionnaire assessing global disability. *Functional Assessment of Chronic Illness Therapy-Fatigue (FACIT-F)* evaluates physical and functional consequences of fatigue in performing daily activities [37].

### 2.5. Statistical Analysis

Descriptive statistics are provided as mean ± standard deviation for continuous variables and as number and percentages for binomial variables. Continuous variables were tested with the Kolmogorov-Smirnov test for normality. In SSc patients and controls, student's *t*-test was used to compare scores of HADS-A and HADS-D and to evaluate scores of HADS-D in subjects with anxiety (scoring ≥8 at HADS-A) in respect of those without anxiety; percent of depressive and anxious symptoms were compared with Fischer exact test. Clinical, clinimetric, and anthropometric characteristics of lSSc and dSSc groups were compared by Fisher exact test or *X* ^2^ test for binomial variables and *t* test and Mann-Whitney test for continuous variables, when appropriate. For correlations of HADS-D and HADS-A scores with clinical, clinimetric, and anthropometric characteristics Spearman's or Pearson correlations tests were used, when appropriate. Variables shown as significantly correlated with HADS-D and HADS-A were entered in hierarchical multiple regression models with HADS-D and HADS-A scores as the dependent variables. For all statistical tests, the threshold of significance was set at a *P* value < 0.05. All analyses were performed using SPSS version 20.0 for Windows (SPSS, Chicago, IL, US).

## 3. Results 

The clinical, clinimetric, and anthropometric measures of SSc patients are presented in Table 1.

### 3.1. HADS Scores

In SSc group, HADS-D (6.14 ± 3.97) and HADS-A (6.66 ± 4.09) scores were higher than in healthy controls (4.72 ± 2.88 and 5.16 ± 3.05) (*P* < 0.05 for both comparisons). 

Basing on a cutoff score ≥8 in both HADS subscales [36], both rates of depression and anxiety in 6 of the whole SSc group were 36%. Fifteen out of 119 patients (13%) had only depression, 15/119 (13%) presented only anxiety, and 28/119 (23%) had both depression and anxiety. 

Depressive patients with comorbid anxiety had significantly higher HADS-D score than patients with depression only (11.39 ± 1.57 versus 9.4 ± 1.64; *P* = 0.001). In healthy controls, the rates of depression and anxiety were 10% (5/50) and 20% (10/50), lower than in SSc (*P* < 0.001 for HADS-D and <0.05 for HADS-A).

In controls, the copresence of depression and anxiety, found in 2/50 subjects (4%), was lower than in SSc (*P* = 0.001).

In controls, depressive subjects with comorbid anxiety had significantly higher HADS-D score than subjects with depression only (9.5 ± 2.1 versus 4.92 ± 2.86; *P* = 0.001).

### 3.2. Comparison between lSSc and dSSc (Table 1)

No significant difference in HADS-D, HADS-A, RSES, COPE NIV subscales, and COPE NIV total score between lSSc and dSSc was found. Hand and mouth disability, global disability, and fatigue were significantly higher and hand and mouth mobility significantly lower in dSSc than in lSSc (*P* < 0.05 for all comparisons).

### 3.3. Correlations of Symptoms of Depression and Anxiety with Psychological, Physical Variables. Global, Hands Disability, and Face Involvement

*HADS-D* was significantly positively correlated with COPE-NVI AS (*P* = 0.001), right and left HAMIS (*P* = 0.013 and 0.020, resp.), FACIT-F, HAQ, MHISS total, MHISS mouth opening, MHISS sicca syndrome, MHISS aesthetic concerns, and CHFDS (*P* < 0.001 for all variables) and negatively with RSES (*P* < 0.001) and mouth opening (*P* = 0.16). 

*HADS-A* was significantly positively correlated with FACIT-F (*P* < 0.001), COPE-NVI AS (*P* < 0.001), HAQ (*P* = 0.001), MHISS total (*P* < 0.001), MHISS mouth opening (*P* < 0.001), MHISS sicca syndrome (*P* = 0.001), MHISS aesthetic concerns (*P* = 0.004), and CHFDS (*P* = 0.032) and negatively with RSES (*P* < 0.001) and COPE-NIV total score (*P* = 0.01). No correlation was found between HADS-A and HADS-D and other variables (Table 2).

### 3.4. Hierarchical Multiple Linear Regression

In SSc patients, by the hierarchical multiple linear regression models, *HADS-D* was significantly associated with FACIT-F (*B* = 0.42; *t* = 5.38; *P* < 0.001), RSES (*B* = −0.29; *t* = −3.62; *P* < 0.001), and MHISS total score (*B* = 0.20; *t* = 2.64; *P* = 0.016), which, together, explained 50.3% of the variance in HADS-D score (FACIT-F: 38.2%; RSES: 9.7%; MHISS total score: 2.4%). 

*HADS-A *was significantly associated with RSES (*B* = −0.24; *t* = −2.79; *P* = 0.006), COPE-NIV SA (*B* = 0.25; *t* = 3.08; *P* = 0.003), COPE-NIV SS (*B* = 0.20; *t* = 2.70; *P* = 0.008), FACIT-F (*B* = 0.20; *t* = 2.33; *P* = 0.022), and MHISS mouth opening (*B* = 0.18; *t* = 2.21; *P* = 0.029). Together, these variables explained 41.2% of the variance in HADS-A score (RSES: 23.9%, COPE-NIV SA: 7.5%, COPE-NIV SS: 3.9%, FACIT-F: 3.7%, and MHISS mouth opening: 2.2%).

## 4. Discussion

Depression and anxiety, frequent in patients with rheumatic diseases, are associated in SSc with impairment of QoL and sexual functioning, nonadherence to treatment regimes, and increased request for healthcare [7, 38, 39]. 

In SSc patients, our results indicate rates of depression and anxiety (36% each) higher than healthy controls (whose rates of depression and anxiety were 10% and 20%, resp.). These data are in agreement with previous results obtained by HADS in SSc [16] and higher than in European general population, where depression and anxiety range from 4.6 % to 23% and from 9.6% to 21%, respectively [40, 41]. 

As far as we know, comorbidity between anxiety and depressive conditions was never evaluated in SSc patients. In our study, it was present in 23% of SSc patients, thus very close to the rate already demonstrated in general population and in other patient groups [16, 17]. We also found that depressive patients with comorbid anxiety present significantly higher HADS-D scores than those with depression only, in agreement with data present in other patients groups [16, 17]. Concordantly with previous studies, no difference in depressive and anxiety symptoms between lSSc and dSSc was found, suggesting that moods disorders may be present in SSc independently from the disease subsets [15]. 

In our study, depressive symptoms were significantly correlated with global disability, hands and mouth disability, fatigue, self-esteem, and avoidance strategy in coping. 

Anxiety was correlated with self-esteem, high level of fatigue, avoidance strategy, social support, and with global, face, and hands disability. 

In SSc, only few studies assessed factors independently related to anxiety and no studies assessed impact of hands and face disability on anxiety [13, 14, 18, 21]. 

The significant correlation between depression and anxiety with hands disability may indicate that an increased need of help in daily life occupations or the experiencing of helplessness could favor the development of depressive and anxiety disorders in SSc patients. 

In agreement with data shown in RA, [38], in SSc, depressive and anxiety symptoms correlate with coping avoidance strategy. In SSc patients, the modifications of the most visible and socially relevant body parts, such as hands and face, may lead to difficulty in social relationships and, consequently, to avoidance behaviors and social anxiety [42]. 

Our data show a direct correlation between the social support and anxiety. Previous studies on the relationship between social support and anxiety provided contradictory results. Some works indicated that individuals with less perceived social support scores reported higher levels of anxiety [43, 44], while other surveys [45] did not find any correlation between social support and anxiety or found a negative effect of social support [46]. Further studies are needed to explore such correlation and to identify the aspects of the social support which may impact negatively on anxiety. 

The correlation of fatigue both to anxiety and to depression is not surprising. In fact, the symptom, that is frequent and mainly due to muscle-skeletal involvement [47, 48], may lead to anxiety and depression by diminishing mobility, independence, and ability to engage in social activities. 

In our patients, self-esteem, correlated to anxiety and depression, may be potentially impaired both by the reduction of physical functioning, causing difficulties in performing daily activities, and by the changes in face and hands [49]. In women with SSc, self-esteem pertaining to appearance was found lower than in burn victims and related to depressive and anxious symptoms [50, 51]. Hierarchical multiple regression analyses revealed that fatigue, firstly, self-esteem, and mouth disability were independent related to depression. Self-esteem, and, secondarily, avoidance strategy, social support, fatigue, and mouth disability were independently associated with anxiety.

Previous studies examining variables related to depressive symptoms did not include the analysis of self-esteem and district disability. These surveys found younger age, digital ulceration, global disability, aspects of personality [6], social support [6, 9], helplessness [8, 9], sense of coherence [8], pain, fatigue, emotional-focused coping, and fear of progression [9] to be independently associated with depression [6, 8, 9]. 

These works, moreover, did not evaluate anxiety together with depression. Our study is the first evaluating bivariate association of district and global disability and psychological variables with anxiety in SSc and using stepwise regression analyses to identify factors independently related to anxiety. 

A limitation of our study is the cross-sectional design, that does not allow us to draw conclusions about prospective association between disease-related functional impairment and psychological distress among SSc patients. 

HADS is a reliable and valid test to screen for depression and anxiety, easy to be used and scored, able to exclude somatic items [52, 53], and potentially useful in everyday practice to assess SSc subjects. Patients resulting positive for the screening should be addressed to a specialist, undergo a more structured clinical interview for further assessment, and be treated by psychoeducational or pharmacological interventions.

Some interventions may be useful to reduce SSc-related distress. Social interaction skills training programs [54], cognitive behavioral therapy for social anxiety [55], and educational and psychotherapy interventions [56] reduced social avoidance and increased self-esteem in social setting for other patients groups and decreased pain, disability, fatigue, and physician visits in patients with other rheumatic diseases. Thus, they could be expected to have similar effects in SSc as well, both if used alone or in combination with pharmacological therapy.

### 4.1. In Conclusion

In SSc patients, depression and anxiety are common and are correlated both with local and global disabilities and to psychological characteristics. By multivariate analysis, depressive symptoms are associated with fatigue, and self-esteem and avoidance strategies of coping are associated with anxiety. SSc patients with depression associated with anxiety have higher level of depressive symptoms. Thus, their treatment should be focused both to depressive and anxiety components. However, future research is needed to confirm HADS as a useful instrument of routine screening for depressive and anxiety disorders and to evaluate the impact of treatment and psychotherapy interventions on psychological distress in SSc patients.

## Figures and Tables

**Table 1:** Clinical, clinimetric, and anthropometric characteristics of SSc patients.

	SSc	lSSc	dSSc	*P* value (lSSc versus dSSc)
Age (years)	59.92 ± 13.49	60.28 ± 13.25	59.31 ± 14.01	NS
Disease duration (years)	10.58 ± 7.42	10.28 ± 8.06	11.05 ± 6.39	NS
HADS-D	6.14 ± 3.97	5.96 ± 3.89	6.44 ± 4.11	NS
HADS-A	6.66 ± 4.09	6.57 ± 3.84	6.80 ± 4.11	NS
RSES	21.30 ± 4.20	21.23 ± 4.16	21.42 ± 4.31	NS
COPE NVI SS subscale	24.46 ± 7.15	24.88 ± 6.95	23.82 ± 7.47	NS
COPE NVI AS subscale	22.02 ± 5.06	22.26 ± 5.14	21.64 ± 4.97	NS
COPE NVI PA subscale	29.90 ± 6.95	30.13 ± 6.93	29.55 ± 7.06	NS
COPE NVI PS subscale	24.92 ± 5.64	24.72 ± 5.22	25.23 ± 6.30	NS
COPE NVI TO subscale	24.12 ± 5.28	23.76 ± 4.90	24.66 ± 5.84	NS
COPE NIV total score	127.97 ± 28.83	129.97 ± 34.23	124.89 ± 17.42	NS
Right HAMIS	5.19 ± 5.36	3.82 ± 4.62	7.40 ± 5.77	<0.001
Left HAMIS	5.03 ± 5.12	3.58 ± 4.08	7.40 ± 5.77	<0.001
CHFDS	10.68 ± 13.10	9.60 ± 15.36	12.51 ± 11.25	<0.05
Right hand opening	3.40 ± 1.34	3.65 ± 1.29	3.01 ± 1.34	<0.05
Left hand opening	3.28 ± 1.38	3.49 ± 1.44	2.95 ± 1.24	<0.05
Right fist closure	0.79 ± 1.40	0.53 ± 1.17	1.19 ± 1.61	<0.01
Left fist closure left	0.82 ± 1.42	0.51 ± 1.19	1.30 ± 1.62	<0.001
MHISS total	18.12 ± 11.21	16.26 ± 11.13	21.18 ± 10.77	<0.05
MHISS mouth opening	8.14 ± 6.09	6.91 ± 631	10.18 ± 5.18	0.001
MHISS Sicca syndrome	6.96 ± 5.26	6.92 ± 4.91	7.02 ± 5.85	NS
MHISS aesthetic concerns	3.43 ± 2.93	3.08 ± 3.00	4.00 ± 2.76	NS
Mouth opening	3.89 ± 0.10	4.14 ± 0.88	3.47 ± 1.04	0.001
HAQ	0.63 ± 0.65	0.55 ± 0.70	0.76 ± 0.53	<0.05
FACIT-F	14.08 ± 9.62	12.77 ± 9.58	16.20 ± 9.41	<0.05

Legend: values are presented as mean ± SD; SSc: systemic sclerosis; NS: not significant; HADS: Hospital Anxiety and Depression Scale; RSES: Rosenberg Self-Esteem Scale; COPE NIV: Coping Orientation to Problems Experienced-New Italian Version (SS: social support subscale; AS: avoidance strategies subscale; PA: positive attitude subscale; PS: problem solving subscale; TO: transcendent orientation subscale); HAMIS: Hand Mobility In Scleroderma Test); CHFDS: Cochin Hand Functional Disability Scale; MHISS: Mouth Handicap in Systemic Sclerosis Scale; HAQ: Health Assessment Questionnaire; FACIT-F: Functional Assessment of Chronic Illness Therapy-Fatigue Scale.

**Table 2:** Correlations of HADS-D and HADS-A with psychological and physical variables.

	HADS-D		HADS-A	
	*P* value	*r*	*P* value	*r*
Age (years)	NS	—	NS	—
Disease duration (years)	NS	—	NS	—
RSES	<0.001	−0.555	<0.001	−0.483
COPE NIV SS subscale	NS	—	<0.05	0.291
COPE NIV AS subscale	0.001	0.304	<0.001	0.494
COPE NIV PA subscale	NS	—	NS	—
COPE NIV PS subscale	NS	—	NS	—
COPE NIV TO subscale	NS	—	NS	—
COPE NIV total score	NS	—	0.01	−0.243
HAMIS right	0.01	0.228	NS	—
HAMIS left	<0.05	0.213	NS	—
HAMIS mean	0.01	0.223	NS	—
CHFDS	<0.001	0.338	<0.05	0.199
Right hand opening	NS	—	NS	—
Left hand opening	NS	—	NS	—
Right fist closure	NS	—	NS	—
Left fist closure	NS	—	NS	—
MHISS mouth opening	<0.001	0.390	<0.001	0.328
MHISS Sicca syndrome	<0.001	0.418	0.001	0.292
MHISS aesthetic concerns	<0.001	0.317	<0.05	0.259
MHISS tot	<0.001	0.482	<0.001	0.365
Mouth opening	0.01	−0.228	NS	—
HAQ	<0.001	0.406	0.001	0.310
FACIT	<0.001	0.616	<0.001	0.440

Legend: values are presented as mean ± SD; SSc: systemic sclerosis; NS: not significant; *P* and *r* values are presented as Pearson's correlation.

HADS: Hospital Anxiety and Depression Scale; RSES: Rosenberg Self-Esteem Scale; COPE NIV: Coping Orientation to Problems Experienced-New Italian Version (SS: social support subscale; AS: avoidance strategies subscale; PA: positive attitude subscale; PS: problem solving subscale; TO: transcendent orientation subscale); HAMIS: Hand Mobility In Scleroderma Test; CHFDS: Cochin Hand Functional Disability Scale; MHISS: Mouth Handicap in Systemic Sclerosis Scale; HAQ: Health Assessment Questionnaire; FACIT-F: Functional Assessment of Chronic Illness Therapy-Fatigue Scale.
